# Reduced Expression of Enac in Placenta Tissues of Patients with Severe Preeclampsia Is Related to Compromised Trophoblastic Cell Migration and Invasion during Pregnancy

**DOI:** 10.1371/journal.pone.0072153

**Published:** 2013-08-19

**Authors:** Shan Wang, Guolin He, Yue Yang, Ying Liu, Ruiying Diao, Kai Sheng, Xinghui Liu, Wenming Xu

**Affiliations:** 1 Department of Obstetrics and Gynecology, West China Second University Hospital, Sichuan University, Chengdu, China; 2 Joint Laboratory of Reproductive Medicine, Sichuan University-The Chinese University of Hong Kong, West China Second University Hospital, Sichuan University, Chengdu, China; 3 Key Laboratory of Obstetric and Gynecologic and Pediatric Diseases and Birth Defects, Ministry of Education, West China Second University Hospital, Sichuan University, Chengdu, China; 4 Shenzhen Key Lab of Male Reproduction and Genetics, Peking University Shenzhen Hospital, Shenzhen, PR China; State Key Laboratory of Reproductive Biology, Institute of Zoology, Chinese Academy of Sciences, China

## Abstract

The purpose of the study is to investigate the expression of epithelial sodium channel (ENaC) in normal pregnancy and severe preeclampsia placenta and to explore the underlying mechanism of the relationship between the altered ENaC expression and onset of preeclampsia. Reverse transcription polymerase chain reaction (RT-PCR) and Western blot were used to check epithelial sodium channel subunits expression in mRNA and protein level in first term and full term placental tissue. ENaCα specific RNAi were used to knockdown ENaC expression and cell invasion and migration assay were used to check whether reduced expression of ENaC can compromise trophoblast cell function. The result showed that ENaCα was highly expressed in first term placental trophoblast cells; while EnaCβ was highly expressed in full term placenta. Knockdown ENaCα expression by using small interfering RNA reduced the invasive and migration abilities of HTR-8/SVneo cell. Real time-PCR and Western blot analysis showed that the expression levels of ENaCβ were also significantly lower in severe preeclampsia compared with normal pregnancy. It is concluded that the ENaC played an important role in trophoblast cell invasion and migration. Reduced expression and activity of epithelial sodium channel in trophoblast cells may be involved in the pathogenesis of preeclampsia.

## Introduction

Preeclampsia, which is characterized by hypertension developed from 20 weeks of gestation, is a serious pregnancy-related disease threatening maternal and children’s health. Although the etiology remains elusive, it is well-accepted that the placenta plays central roles in the pathogenesis of precclampsia, since removal of the plancenta by abortion or delivery is the only effective treatment for preeclampsia [1], [2]. Placenta is an organ supporting normal pregnancy and ensure normal embryonic, fetal development; on the other hand, poor placentation has been associated with preeclampsia since remodeling of spiral arteries is often incomplete as assessed by Doppler ultrasonography in these patients [1].

Poor placentation can result from insufficient placenta trophoblastic cell invasion and differentiation [2]. The current hypothesis holds that shallow invasion and poor differentiation of the trophoblast impede spiral arteries remodeling, resulting in restricted blood supply and hypoxia in the maternal-placenta interface. There are also evidences showing that the imbalance of pro-angiogenic and anti-angiogenic factors that released from the placenta contributing to the onset of the disease and correlated to the disease severity. Therefore, understanding the mechanism of how trophoblastic cells invasion and differentiation is regulated at molecular level may contribute to the understanding of the pathophysiology of preeclampsia.

The ENaC is an ion channel highly expressed in epithelia cell of the lung, kidney, brain and reproductive tract. It is believed that ENaC is composed of three structurally related subunits, α, β, and γ, possibly with a α1:β1:γ1 subunit stoichiometry [3]. The major function of ENaC is absorption of sodium and therefore responsible for transepithelial ion transportation. The importance of ENaC in reabsorption function of renal collection duct cells has been exemplified by the correlation of ENaC expression with salt-sensitive high blood pressure [4]. R536Q mutation of ENaC β subunit was detected in patients of Liddle’s syndrome during pregnancy, raising the possibility that ENaC may also play roles in pregnancy related hypertension [5]. Interestingly, ENaC is expressed in the placenta trophoblastic cells [6]. A recent study showed reduced expression of ENaC in full term placentas in preeclampsia compared to normal pregnancy, suggesting that ENaC may play a role in the pathogenesis of preeclampsia [7]. Furthermore, inhibition or knockdown of ENaC α subunit was found to inhibit the migration of the BeWo, a human trophoblastic cell line [8], indicating a role of ENaC in trophoblastic cell migration and placentation. However, whether ENaC expression is related to onset of preeclampsia is still unexplored. Our study shows that decreased epithelial sodium channel expression was detected in preeclampsia placenta compared with normal pregnancy. Furthermore, dysfunction of epithelial sodium channel may affect normal trophoblast invasion and migration, which have been shown to be important for normal placental development.

## Materials and Methods

### The Object of Study and Diagnostic Criteria

The study was approved by the ethics committee of West China Second University Hospital of Sichuan University and all the consent forms were signed. All the subjects we chosen were in March 2009 ∼ May 2011 in our hospital with cesarean section for pregnant women whose termination weeks of pregnancy are between 35 weeks and 39 weeks. Preeclampsia was defined as systolic blood pressure of >140 mmHg and/or diastolic blood pressure of >90 mmHg on two occasions >6 h apart after 20 weeks of gestation before the onset of labor, plus proteinuria of >2+ (dipstick method) or >0.3 g/24 h (American College of Obstetricians and Gynecologists, 1996). Severe preeclampsia was defined as a higher blood pressure >160 mmHg systolic or >110 mmHg diastolic on two occasions >6 h apart, and a proteinuria level >5 g/24 h or >3+ by dipstick testing on at least two separate occasions (American College of Obstetricians and Gynecologists, 1996). The gestation-matched placentas of normal pregnancy were chosen as normal control. The two groups of pregnant women were singleton pregnancy, and excluded patients with previous heart, liver, kidney, thyroid, diabetes history. A total of 44 control and 48 patients were recruited in the study.

### Specimen Collection

Two groups of subjects were chosen and the placenta were cut after cesarean section in maternal side, taking tissue around the central villus area, rinsed with saline and quickly placed in liquid nitrogen, and put in −80°C refrigerator for frozen reserve. The first term placentae (8–11 weeks amenorrhea) obtained following elective abortion carried out by vacuum aspiration at the University Hospital, in accordance with ethical guidelines of West China Second University Hospital. One part of fresh placentae were fixed in 4% PFA and processed to immunohistochemical staining. For the RNA and protein study, term villus tissues from the middle of placenta were cut in maternal side and a piece of tissue about 1*1*1 cm in size were saved, avoiding blood clot, infarction and calcification parts; Both RNA and protein lysates were prepared from it.

### Immunohistochemistry

Immunohistochemical staining was carried out according to conventional operation SP kit (VECTASTAIN® ABC kit, Vector labs, USA) with minor revision. Briefly, 5-µm sections were sequentially cut and mounted onto gelatin-treated slides. Then they were dried overnight at 37°C. After that, all slides were deparaffinized in xylene and rehydration through graded ethanol. In order to retrieve the epitope, slides were immersed in a boiled water bath for 20 minutes with citrate buffer at pH 6.0. The slides were cooled down at room temperature for 20 minutes, and H_2_O_2_ (0.3%) were incubated on slides at room temperature for 20 minutes. After serum blocking, primary antibodies were added at 4°C overnight. β-ENaC rabbit anti-human polyclonal antibody (l∶200, abcam company, UK) and α-ENaC (1∶100, Cat No:10924-2-AP, Proteintech, China), γ-ENaC (1∶100, Cat No:13943-1-AP, Proteintech, China), were used as first antibodies for IHC staining, respectively. PBS buffer instead of primary antibody were used as blank control. Immmuohistochemical results were analysed by light microscopy (TI-U, Nikon CLEIPSE, NIKON, Japan ) with Camera (SPOT Flex™ Camera, Diagnostic Instrument, USA). The *SPOT advance* software was used for image analysis.

### Real Time PCR

Total placenta RNA was prepared from frozen placenta tissues with Trizol reagent (Invitrogen). Genomic DNA contamination was removed by performing Dnase I digestion for 10 min at RT. Total RNA was eluted in DEPC treated water. Samples were considered for further analysis when the OD260/280 ratio was between 1.8 and 2.0. cDNA was synthesized using SuperScript II Reverse Transcriptase (Invitrogen). No reverse transcriptase controls were prepared for each sample. Standard curves for each target were prepared from pooled samples of first-strand cDNA from samples. Reaction efficiencies were determined form the standard curves and the results were calibrated with amplification efficiency using CFX Manager 2.0 software (Bio-Rad, USA). Quantitative real-time PCR (qRT–PCR) was performed using a Bio-Rad CFX96 system (Bio-Rad, USA) and iTaq™ SYBR green supermix (Bio-Rad). RNA concentration was adjusted to 1 µg/ul. α-ENaC primers: The upstream primer sequence: 5′-AACTATCGCACCATTGAAGAATC-3′; downstream primer: 5′-ATGAGGCACAGCACAGAG -3′. Β-ENaC mRNA amplification product length of 189 bp. α-ENaC primers: The upstream primer sequence CTGGAAGGACTGGAAGAT; downstream sequence: GGATGTTGATGTAGTGGAAG; γ-ENaC primers: The upstream primer sequence: TCCTCGTCTTCTCCTTCTA; downstream sequence: GTCTCCTGTTCCAAGTCA. GAPDH primer sequences: upstream primer sequence: 5′-TGCACCACCAACTGCTTAGC-3′; downstream primer: 5′-GGCATGGACTGTGGTCATGAG -3′ were used as housekeeping gene for normalization. Water was used as negative controls in every amplifications.

### Western Blot

Placenta tissue were lysed in RIPA buffer (150 mM sodium chloride; 1.0% NP-40 OR Triton X-100; 0.5% sodium deoxycholate; 0.1% SDS; 50 mM Tris, PH8.0) with manual extraction of placenta total protein, BCA method was used to quantify the protein concentration. β-ENaC rabbit anti-human polyclonal antibody (l∶200, abcam company, UK) and α-ENaC (Cat No:10924-2-AP, Proteintech, China) was used as 1stAb and HRP labeled goat anti-rabbit IgG (1∶5000, KPL Inc, China) were used as 2nd Ab. The membrane was detected with chemiluminescence kit (Cat. Num:WBKLS0500, Millipore company, USA) and the signal was developed in the gel imaging system.The strength of bands were analyzed with quantity one software (Bio-Rad), and the corresponding internal reference GAPDH (1∶3000, Kangchen BIO-TECH Corporation, Shanghai, China) or β-tubulin (Zen BioScience, Chengdu, China) was used as internal control.

### Cell Culture and Transfection

Human extravilloustrophoblast(EVT) cell line, HTR- 8/SVneo, was incubated in RPMI 1640 medium supplemented with 10% fetal bovine serum at 37°C in a 5% CO_2_ atmosphere. The epithelial sodium channel sequence-specific small interfering RNA (SiRNA/ENaC) and scrambled control RNA (SiRNA/ctrl) were synthesized by Ribobio company (Guangzhou, China) and transfected into HTR-8/SVneo cells with lipofectamine™ 2000 reagent. The sequence of SiRNA was synthesized by Guangzhou RIBOBIO Company. 6-well matrigel gel coated plates (Cat.Number: 356231, BD Bioscience, USA) or 24-well transwell cell culture chambers (Part number: 70019, CHEMICON international, USA) were used for the migration and invasion assays, respectively.

We transfected HTR-8/siRNA and scrambled negative control RNA to HTR-8/SVneo cells at 50 nmol/L concentration, transfected concentration gradient were set as 50 nM, 20 nM, 10 nM respectively, cells that were cultured under the same conditions were tested by a Western blot analysis 48 hours later. Finally we choose 50 nmol/L concentration as the most effectively transfecting concentration, and the migration and invasion experiment were done after 48 hr’s transfection.

### Migration Assay

Two groups called SiRNA/Nctrl and SiRNA/ENaC were prepared as above, wound was created by manually scraping the cell monolayer with a p200 pipet tip, marking on the outer bottom of the dishes to be used as reference points during image acquisition, and then incubated dishes in a tissue culture incubator for 48 hours. Finally the images were acquired through the microscope by photographing.

### Invasion Capability Assay

Preparing a cell suspension containing 0.5–1.0×10^6^ cells/ml which had been transfected by ENaC siRNA and control respectively. One group of empty transfection was used to eliminate non-specific effect of transfection. 300 ul of prepared cell suspension was added to each insert and added 500 ul of media containing 10% fetal bovine serum to the lower chamber of 24-well plate. Incubating cells for 72 hours in a tissue culture incubator and then stained invasive cells with staining solution. The procedure for preparing staining solution is as follow: crystal violet were dissolved in methanol in 0.5% stock solution and further diluted with PBS in 1∶4 dilutions for staining. Finally the cells were counted by photographing the membrane through the microscope. The number of migrated cells was counted at ×100 magnification.

### Statistical Analysis

The data were analyzed by Statistical package SPSS15.0. Data were presented as mean ± SEM in the figure. T test was used for two sample test of homogeneity of variance between groups of ENaC mRNA and protein. For three groups, one way ANOVA was used for the comparison. P<0.05 was used as statistical difference.

## Results

### Expression of and Hormone Regulation of ENaC in Trophoblast

**Figure 1:**
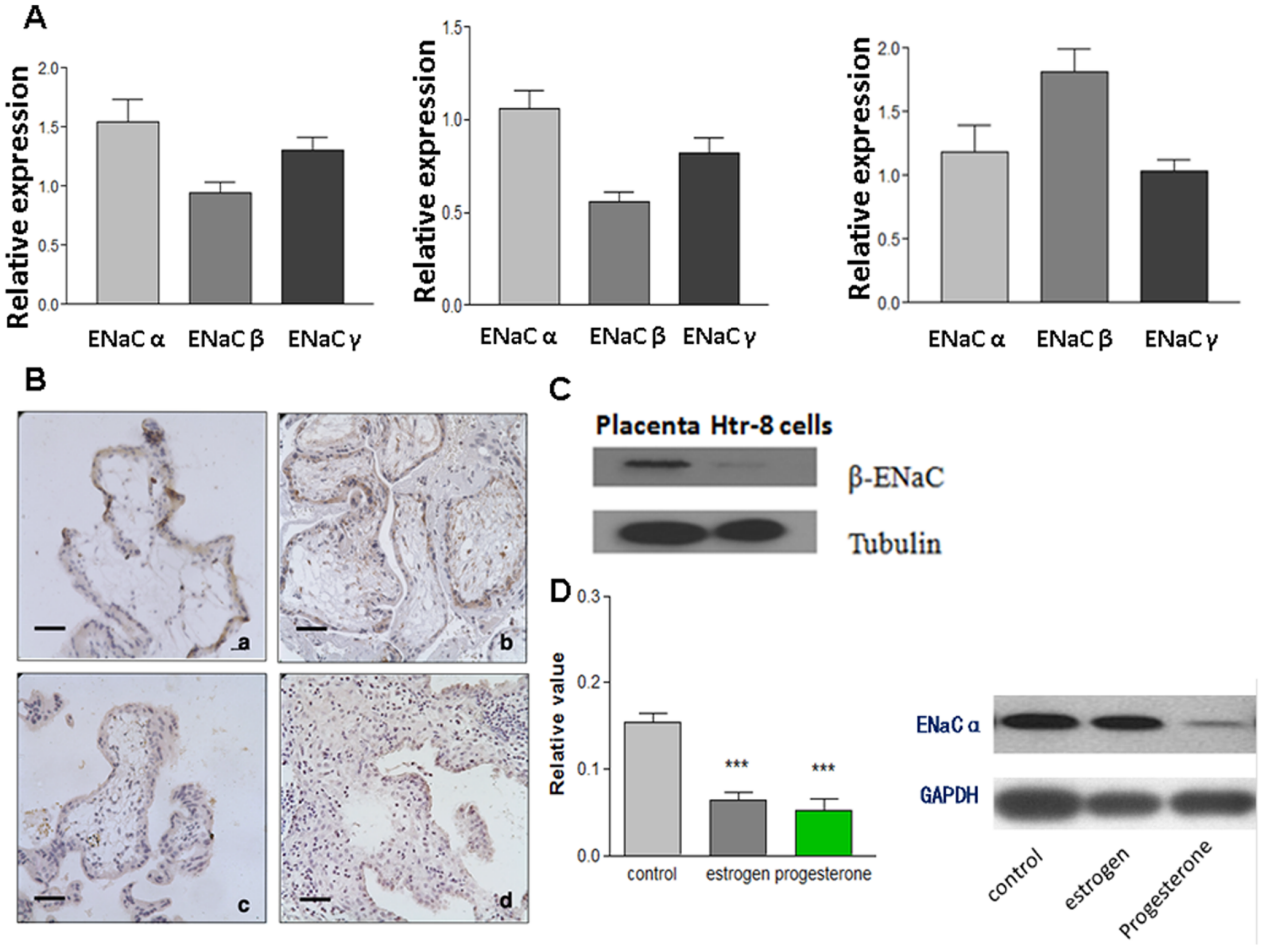
Expression of ENaC subunits in the first term and full term placenta. (**A**) real time-PCR result of the ENaC subunits expression in first term villous (left pannel), decidue tissues (middle pannel) and full term placenta (right pannel). α-ENaC is more abundenly expressed on the first term villous and decidual cells; while β-ENaC is more abundenly expressed on the full term villous. (**B**) The immunohistochemistry staining of α-ENaC in first term villous (a,c) and third term placenta of normal pregnancy(b,d). (**a**) The positive staining of α-ENaC is on the apical plasma membrane of the syncytiotrophoblast. Villous cytotrophoblastic cells are also stained. (**b**) Immunostainingof α-ENaC from the third term placenta. Very weak staining was seen in the syncytiotrophoblast cells. (c,**d**) negative control with normal IgG in first term and third term placenta tissues. Magnification (X400). Scare Bar = 40 µM. (**C**) Western blot shows that HTR-8 cell express low level of β-ENaC, although high level of β-ENaC expression could be detected on full term placenta. (**D**) The effects of progesterone, estrogen on the α-ENaC expression in HTR-8/SVneo cells. Left pannel: HTR-8/SVneo cells were treated with progesterone (5 µM), estrogen (1 µM) respectively for 24 hr, and real time PCR was used to check α-ENaC expression. Relative expression levels between different groups were compared. The result shows that progesterone and estrogen could inhibited α-ENaC expression. Data were analyzed with one way ANOVA (*means p<0.05; ***means p<0.001). Right pannel: Western blot further confirmed that progesterone (5 µM) could reduce α-ENaC expression.

**Figure 2:**
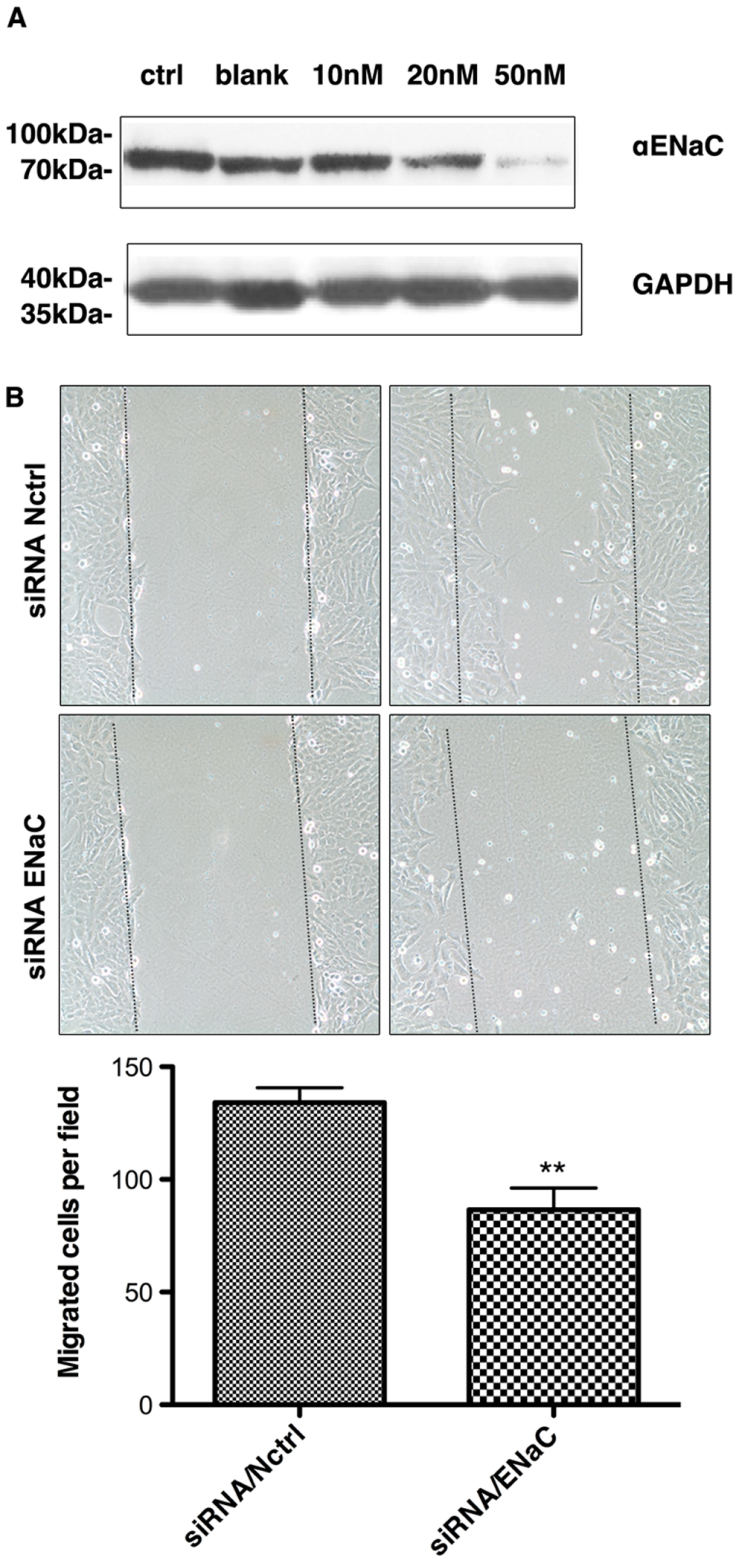
Knowkdown of α-ENaC reduced the migration and invasion capability in HTR8/SVneo cells. (A) Western blot result shows that α-ENaC RNAi can dose-dependently reduce the expression of ENaC. (B) The representative pictures in cell wounding and migration assay. The pictures shows the migration of HTR8/SVneo cells in the martrigel surface after 32 hr of scrape in different groups. The dot line shows the initial start point where the cell began the migration process. The cell number between in dot lines were count. The right is the statistics of the cell migration ability. The data are expressed as means ± SEM, of the three measurements (*means p<0.05).

We first examined the expression of the three subunits of ENaC in human placenta at early and late gestational stage by real time-PCR. The results showed that the expression of α subunit is more abundant in first trimester placenta compared to that of third trimester (Figure 1A; left pannel), The higher expression of α subunit was also observed in decidual tissues from first trimester legal abortion. (8–10 weeks, Figure 1A; middle pannel); while expression of β subunit showed an opposite trend, with higher expression in third trimester placenta than in first trimester (Figure 1A; right pannel). We also examined the expression of ENaC in an extravillous trophoblast cell line from first trimester, HTR-8/SVneo. This cell line has strong expression of α but weak expression of β, which is consistent with the result from the first trimester placenta tissues (Figure 1B, Figure 2A).

It is well known that progesterone and estrogen can modulate ENaC subunit mRNA level in the kidney and lung, respectively [9], indicating the hormone regulation of ENaC could be a general mechanism for modulation of their function. In fact, we found that estrogen (1 µM) or progesterone (5 µM) treatment for 24 h downregulated α-ENaC expression, as demonstrated by quantitative RT-PCR (Figure 1C; left pannel). Western blot further showed that progesterone can inhibit α-ENaC expression in protein level (Figure 1C; right pannel). This is in line with the result of lower expression of ENaCαin third trimester placenta than in first trimester placenta, since estrogen and progesterone levels increase progressively during normal pregnancy and peak at third trimester [10].

### Involvement of ENaC in Trophoblastic Cells Migration and Invasion

The dynamic expression of ENaC in trophoblast during pregnancy suggests that it may play a role in trophoblast function, probably in placentation. Trophoblast migration and invasion into the endometrium are early and prerequisite events of implantation and placentation, therefore we examined whether ENaC is involved in these processes.

We knocked down ENaCα expression with specific siRNA. Western-blot result showed that at concentration of 50 nM, the ENaCα siRNA could significantly down-regulate ENaCα at protein levels (Figure 2A). So we used this concentration for the functional experiments.

Cell monolayer wounding and migration test were performed to examine the role of ENaC on the cell migration. HTR-8/SVneo cells were seeded in 6-well plates. ENaCα was knockdown by 50 nM ENaCα siRNA or negative control siRNA. Forty-eight hours after transfection, wounds were created by scrapping the cell monolayer by P200 pipette tips. As shows in Figure 2B, knockdown of ENaCα by siRNA (siRNA/ENaCα) decreased the migration rate of HRT-8/SVneo cells, as compared to that transfected with negative control siRNA (siRNA/Nctrl), indicating that ENaC is involved in migration capability of trophoblastic cells (Figure 2B).

**Figure 3:**
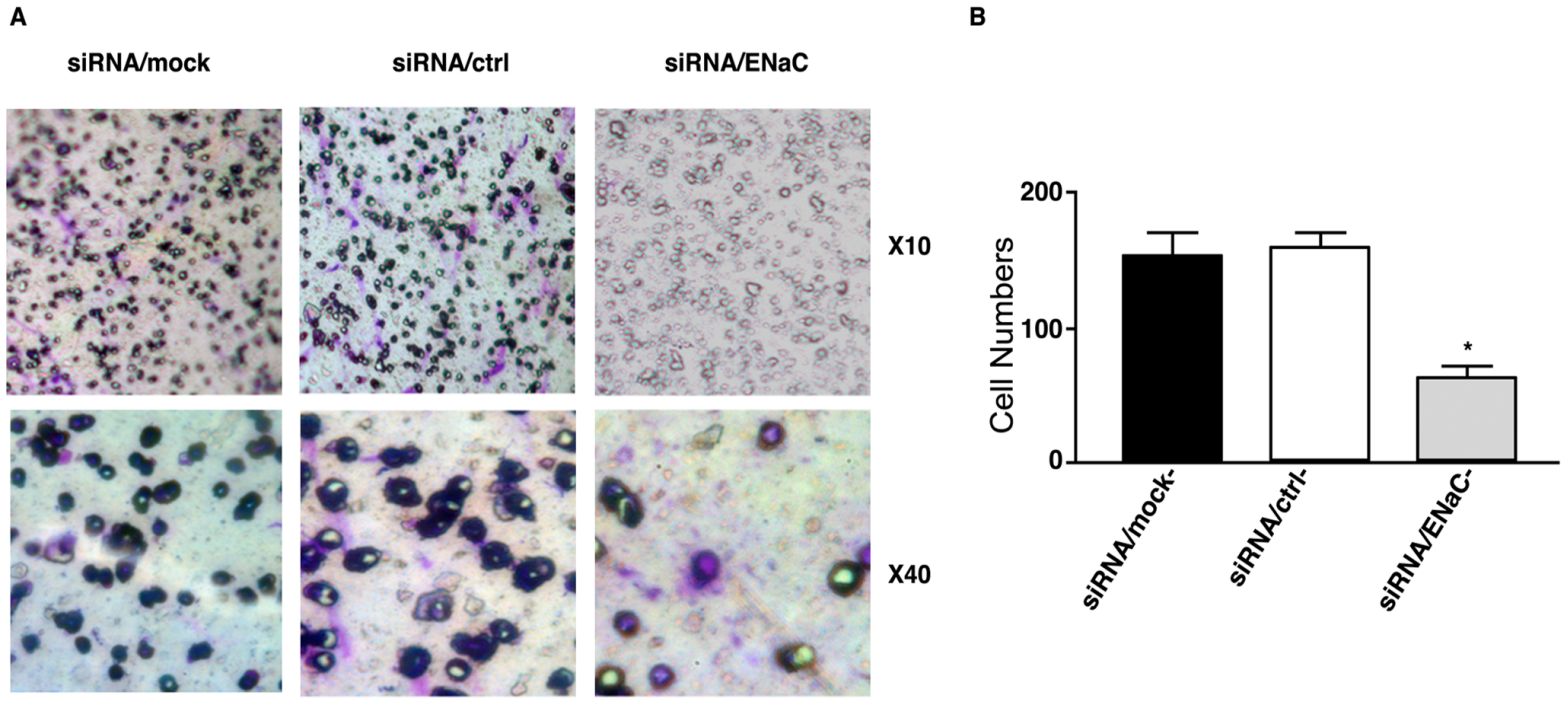
Knowkdown of α-ENaC reduced the invasion capability in HTR8/SVneo cells. (A) is the representative picture of cell invasion assay after 48 hr of invasion assay in the insert of 24 wells. Each group was repeated three times. (B) is the counting result of the cells number under microscope. The cells on the underside of the filters and the pores occupied were counted in 40 randomly selected non-overlapping fields of the membranes under a light microscope at x100 magnification. Student T test were used for comparison. (*meansP<0.05).

Matrigel invasion assay was performed to examine the role of ENaC in trophoblastic cell invasion. HRT-8/SVneo cells were seeded on the upper chamber of transwells coated with matrigel. After 48 hours, the number of cells that invaded to the lower chamber is significantly lower in ENaCα knockdown group (siRNA/ENaCα) compared to the control siRNA group (P<0.05; Figure 3), indicating that knockdown of ENaC suppressed the invasion abilities. The result indicated that the ENaC play an important role in the invasion capability of trophoblastic cells.

The involvement of ENaC in trophoblastic cell migration and invasion suggest a role of ENaC in implantation.

### Reduced ENaC Expression in Preeclamptic Placenta

**Figure 4:**
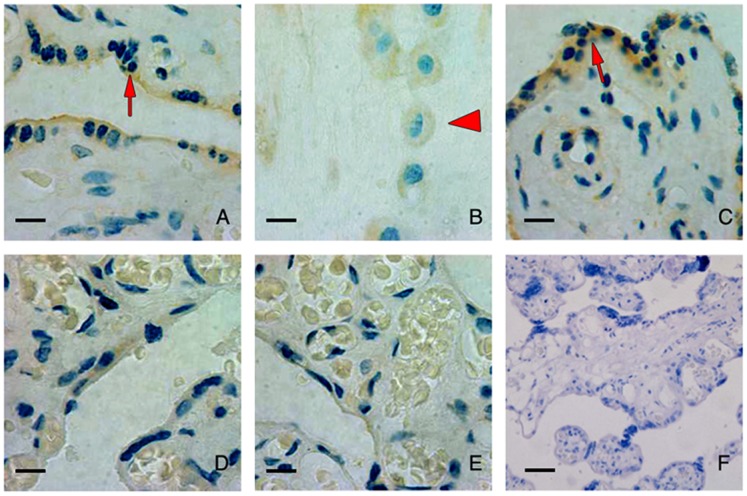
Immunohistochemistry staining of β-ENaC in placenta of normal pregnancy and preeclmpsia patients. The immunohistochemistry staining of β-ENaC in placenta tissue of full-term placenta of normal pregnancy(A-C). The signal was detected in placental villous trophoblast cells, especially in syncytiotrophoblast (arrow, A) with less staining in cytotrophoblast and the staining was seen in extravillous cytotrophoblastic cells (arrowhead, B). (D, E) are staining of β-ENaC in placenta tissue from the preeclampsia patients. Reduced expression was noticed in placenta of preeclampsia patients (N = 3). (F) is negative control of the IHC staining. Magnification: X400, scare bar = 20 µM.

It is believed that shallow implantation and abnormal placentation contribute to the pathogenesis of preeclampsia. Reduction in migration and invasion ability of trophoblastic cells by knockdown of ENaC suggested reduced ENaC expression in trophoblast might be involved in pathophysiology of preeclampsia. We stained β subunits of ENaC in the placenta of severe preeclampsia patients and placenta from normal pregnancy at third trimester. The result showed that the staining intensity of ENaCβ was reduced in the placenta of preeclampsia patients compared to that from normal pregnancy (Figure 4). However, we did not detect positive immuno signals of ENaCα in either preeclamptic or normal placenta at third trimester (data not shown).

**Figure 5:**
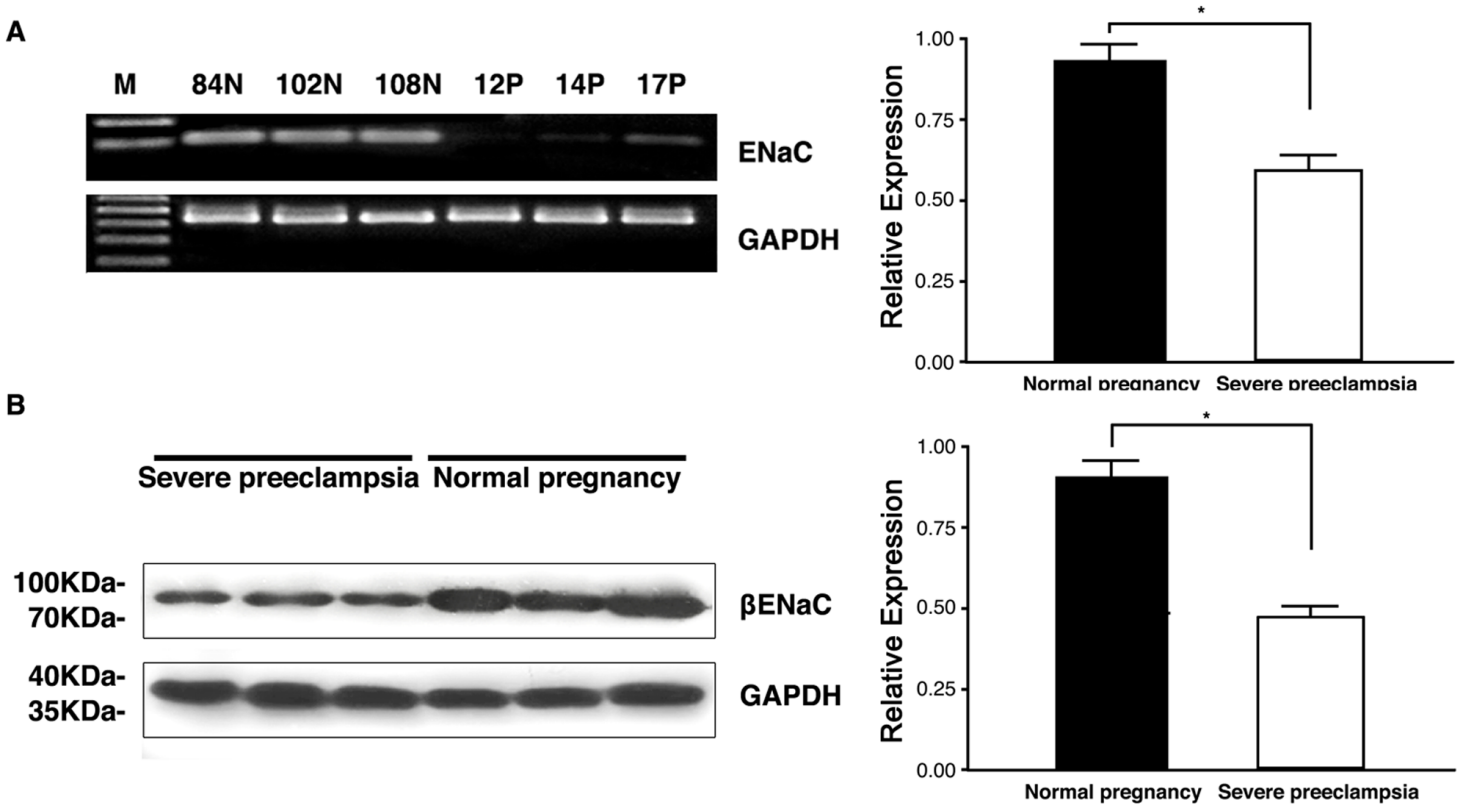
The expression of β-ENaC was significantly reduced in the placenta of preeclampsia patients. β-ENaC expression in placenta of preeclampsia was reduced in RNA and protein level. (A) right bar chart represent the statistics of the real time PCR result, while left figure is the representative gel result of the RT-PCR result of the placenta samples. 12P,14P,17P were patients numbers of severe preeclampsia while left were normal controls. (B) Representative result of the Western blot result; The right is the statistic result (*means p<0.05).

We then used RT-PCR and Western blot to confirm the result of immunostaining in samples obtained from third trimester plancenta from severe preeclampsia patients and normal pregnant women in our hospital. As shown in Figure 5A, Real-time PCR result showed that expression of ENaCβ was significantly lower in placenta from preeclampsia patients (ROD value 0.6021±0.03824, n = 48) than that from normal pregnancy (ROD value 0.9340±0.04686, n = 44; P<0.05; Figure 5A). Western blot results showed that placenta express ENaCβ protein with a molecular mass of 95 kDa protein; expression of ENaCβ protein was significantly lower in placenta from preeclampsia patients (ROD value 0.4848±0.01812, n = 48) than that from normal pregnancy (ROD value 0.9056±0.03886, n = 44; P<0.05; Figure 5B).

We also checked whether the expression of ENaCα were changed in mRNA level in preeclamptic placenta. Though expression of ENaCα was also lower in severe preeclampsia compared with normal pregnancy, there was no statistical difference between two groups (data not shown).

The reduced ENaCβ expression in preeclampsia at mRNA and protein level suggests that deficiency in ENaC subunit expression and possible reduction of the channel function may contribute to the shallow implantation and abnormal placentation in preeclampsia.

## Discussion

Recent studies have found that the ENaC was expressed in placenta trophoblastic cells [6]. In this study, we detected the expression and localization of α, β and γ subunits of ENaC in the placenta from normal pregnancy. Furthermore, we demonstrated that ENaC was localized in the membrane of trophoblast cells and important for HTR-8 cell migration and invation. Rayer et al found that one of ENaCβ mutation called R536Q was related to occurrence of preeclampsia [5]. Using real-time PCR and Western blot, our study showed that the expression of ENaC was reduced in preeclampsia patients compared with that of normal pregnancy, suggesting that ENaC may be involved in the pathological process during the progress of preeclampsia. The question is how the defect of ENaC could contribute to the onset of preeclampsia?

It has long been shown that the depth of placental invasion of endometrial is shallow in preeclampsia patients compared with normal pregnancy [2]. In these patients the trophoblast invasion of uterine spiral arteries stopped at the decidual/myometrial boundary, so the placenta perfusion was in deficiency [2], [11]. The physiological invasion process of the trophoblastic cells was similar to tumor cells, and previous studies have shown that ions like Na^+^, Ca^2+^, K^+^, and Cl^−^ were involved in the tumor cell invasion and differentiation [12]. Our study found that blocking of the ENaC expression using small interfering RNA inhibit invasion and migration ability in trophoblastic cell line, suggesting that the defect of ENaC may compromised trophoblast invasion of uterine spiral arteries in pathological condition, therefore maybe an important etiological factor in the pathogenesis of preeclampsia.

One interesting finding of our study is that we found that of the three subunits constituting ENaC channel, only β subunit were highly expressed in full term placenta and reduced expression were seen in preeclampsia samples, while in first term placenta and trophoblast cells it’s the α, instead of β, were highly expressed. Whether the reduced expression of β subunit is a result from preeclampsia or a cause of preeclampsia is an interesting question. However, since it is impossible to detect β subunit expression in early pregnancy of patients with severe preeclampsia, the question can not be answered conclusively in the current study. Neverthess, the dynamic expression change in ENaC subunits and the involvement of ENaC subunits in trophoblast invasion indicate the role of ENaC in preeclampsia warrant further study. In the kidney, the ENaCα is the most hormone sensitive subunit compared with other subunits, with a hormone responsive element presenting in the promoter of the gene [13]. In placenta, it is highly possible a similar mechanism lies and the increasing high level of progesterone before the labour inhibited the expression of α subunit, while the β subunit is more insensitive to this hormone, therefore more stable during the pregnancy process [9]. We therefore choose α-ENaC specific RNAi to investigate the function of ENaC in first term trophoblast cells invasion, which has been shown important for etiology of preeclampsia. Aldosterone, a well known ENaC regulating hormone is also developmental regulated during pregnancy process [14], [15], whether aldosterone is also involved in the expression switch of subunits of ENaC remain further clarified.

The exact signaling pathway for the involvement of ENaC in trophoblast invasion need further study. Trophoblast invasion and artery remodeling are thought as two major events during embryo implantation process during first trimester. It has been found that many serine protease’s activity were increased in the process of embryo implantation and trophoblast invasion [16], and epithelial sodium channel can also be activated by some of these serine protease like trypsin, furin and CAP [17]. Interestingly, a recent paper shows that deregulation of SGK1, a kinase involved in epithelial ion transport through regulation of ENaC and other ion transporters, could lead the feto-maternal interface being prone to oxidative damage, thus highlight the importance of ion channels in the cross-talk of feto-maternal interface [18]. In this regard, elucidating the molecular mechanism of the involvement of ENaC in this process holds the promise for the better understanding and treatment for preeclampsia and other disorders related to pregnancy.

## References

[1] Fisher S (2004) The placental problem: Linking abnormal cytotrophoblast differentiation to the maternal symptoms of preeclampsia. Reproductive Biology and Endocrinology 2: 53. 15236649 10.1186/1477-7827-2-53 PMC493282

[2] Kaufmann P, Black S, Huppertz B (2003) Endovascular Trophoblast Invasion: Implications for the Pathogenesis of Intrauterine Growth Retardation and Preeclampsia. Biology of Reproduction 69: 1–7. 12620937 10.1095/biolreprod.102.014977

[3] Staruschenko A, Adams E, Booth RE, Stockand JD (2005) Epithelial Na+ Channel Subunit Stoichiometry. Biophysical journal 88: 3966–3975. 15821171 10.1529/biophysj.104.056804 PMC1305628

[4] Bubien JK (2010) Epithelial Na+ Channel (ENaC), Hormones, and Hypertension. Journal of Biological Chemistry 285: 23527–23531. 20460373 10.1074/jbc.R109.025049 PMC2911345

[5] Rayner BL, Owen EP, King JA, Soule SG, Vreede H, et al (2003) A new mutation, R563Q, of the beta subunit of the epithelial sodium channel associated with low-renin, low-aldosterone hypertension. Journal of Hypertension 21: 921–926. 12714866 10.1097/00004872-200305000-00016

[6] del Mónaco S, Assef Y, Damiano A, Zotta E, Ibarra C, et al (2006) Caracterización del canal epitelial de sodio en sinciciotrofoblasto de placenta humana preeclamptica. Medicina (Buenos Aires) 66: 31–35. 16555725

[7] Marino GI, Kotsias BA (2012) Expression of the epithelial sodium channel sensitive to amiloride (ENaC) in normal and preeclamptic human placenta. Placenta. 10.1016/j.placenta.2012.11.008 23218889

[8] del Mónaco S, Marino G, Assef Y, Damiano A, Kotsias B (2009) Cell Migration in BeWo Cells and the Role of Epithelial Sodium Channels. Journal of Membrane Biology 232: 1–13. 19911219 10.1007/s00232-009-9206-0

[9] Gambling L, Dunford S, Wilson CA, McArdle HJ, Baines DL (2004) Estrogen and progesterone regulate [alpha], [beta], and [gamma]ENaC subunit mRNA levels in female rat kidney. Kidney Int 65: 1774–1781. 15086916 10.1111/j.1523-1755.2004.00593.x

[10] Hirota Y, Cha J, Dey SK (2010) Revisiting Reproduction: Prematurity and the puzzle of progesterone resistance. Nat Med 16: 529–531. 20448578 10.1038/nm0510-529 PMC3725962

[11] Lyall F (2005) Priming and remodelling of human placental bed spiral arteries during pregnancy – A Review. Placenta 26: S31–S36. 15837064 10.1016/j.placenta.2005.02.010

[12] Wareing M, Greenwood SL (2011) Review: Potassium channels in the human fetoplacental vasculature. Placenta 32: S203–S206. 21227507 10.1016/j.placenta.2010.12.022

[13] Mick VE, Itani OA, Loftus RW, Husted RF, Schmidt TJ, et al (2001) The α-Subunit of the Epithelial Sodium Channel Is an Aldosterone-Induced Transcript in Mammalian Collecting Ducts, and This Transcriptional Response Is Mediated via Distinct cis-Elements in the 5′-Flanking Region of the Gene. Molecular Endocrinology 15: 575–588. 11266509 10.1210/mend.15.4.0620

[14] Gennari-Moser C, Khankin EV, Schüller S, Escher G, Frey BM, et al (2011) Regulation of Placental Growth by Aldosterone and Cortisol. Endocrinology 152: 263–271. 21068161 10.1210/en.2010-0525

[15] Redman CWG, Sacks GP, Sargent IL (1999) Preeclampsia: An excessive maternal inflammatory response to pregnancy. American Journal of Obstetrics and Gynecology 180: 499–506. 9988826 10.1016/s0002-9378(99)70239-5

[16] Sargent IL, Borzychowski AM, Redman CWG (2007) NK cells and pre-eclampsia. Journal of reproductive immunology 76: 40–44. 17482272 10.1016/j.jri.2007.03.009

[17] Soundararajan R, Melters D, Shih IC, Wang J, Pearce D (2009) Epithelial sodium channel regulated by differential composition of a signaling complex. Proceedings of the National Academy of Sciences 106: 7804–7809. 10.1073/pnas.0809892106 PMC2683131 19380724

[18] Salker MS, Christian M, Steel JH, Nautiyal J, Lavery S, et al (2011) Deregulation of the serum- and glucocorticoid-inducible kinase SGK1 in the endometrium causes reproductive failure. Nat Med 17: 1509–1513. 22001908 10.1038/nm.2498

